# Respiratory Rate Estimation during Walking and Running Using Breathing Sounds Recorded with a Microphone

**DOI:** 10.3390/bios13060637

**Published:** 2023-06-08

**Authors:** Chiara Romano, Andrea Nicolò, Lorenzo Innocenti, Marco Bravi, Sandra Miccinilli, Silvia Sterzi, Massimo Sacchetti, Emiliano Schena, Carlo Massaroni

**Affiliations:** 1 Departmental Faculty of Engineering, Università Campus Bio-Medico di Roma, 00128 Rome, Italy; c.romano@unicampus.it (C.R.); e.schena@unicampus.it (E.S.); c.massaroni@unicampus.it (C.M.); 2 Department of Movement, Human and Health Sciences, University of Rome “Foro Italico”, 00135 Rome, Italy; lorenzo.uniroma4@gmail.com (L.I.); massimo.sacchetti@uniroma4.it (M.S.); 3 Unit of Physical and Rehabilitative Medicine, Università Campus Bio-Medico di Roma, 00128 Rome, Italy; m.bravi@policlinicocampus.it (M.B.); s.miccinilli@policlinicocampus.it (S.M.); s.sterzi@policlinicocampus.it (S.S.)

**Keywords:** wearable sensors, breathing sounds, validation protocol, respiratory frequency, measurement accuracy, exercise, sport sensors

## Abstract

Emerging evidence suggests that respiratory frequency (*f_R_*) is a valid marker of physical effort. This has stimulated interest in developing devices that allow athletes and exercise practitioners to monitor this vital sign. The numerous technical challenges posed by breathing monitoring in sporting scenarios (e.g., motion artifacts) require careful consideration of the variety of sensors potentially suitable for this purpose. Despite being less prone to motion artifacts than other sensors (e.g., strain sensors), microphone sensors have received limited attention so far. This paper proposes the use of a microphone embedded in a facemask for estimating *f_R_* from breath sounds during walking and running. *f_R_* was estimated in the time domain as the time elapsed between consecutive exhalation events retrieved from breathing sounds every 30 s. Data were collected from ten healthy subjects (both males and females) at rest and during walking (at 3 km/h and 6 km/h) and running (at 9 km/h and 12 km/h) activities. The reference respiratory signal was recorded with an orifice flowmeter. The mean absolute error (MAE), the mean of differences (MOD), and the limits of agreements (LOAs) were computed separately for each condition. Relatively good agreement was found between the proposed system and the reference system, with MAE and MOD values increasing with the increase in exercise intensity and ambient noise up to a maximum of 3.8 bpm (breaths per minute) and −2.0 bpm, respectively, during running at 12 km/h. When considering all the conditions together, we found an MAE of 1.7 bpm and an MOD ± LOAs of −0.24 ± 5.07 bpm. These findings suggest that microphone sensors can be considered among the suitable options for estimating *f_R_* during exercise.

## 1. Introduction

Respiratory monitoring is gaining increasing consideration in the field of sports and exercise. Emerging evidence suggests that respiratory frequency (*f_R_*) reflects physical effort and is sensitive to changes in exercise tolerance [1,2,3], with important implications for exercise management in different populations. Indeed, *f_R_* is more closely associated with perceived exertion than other commonly monitored physiological variables (e.g., heart rate), it is influenced by experimental conditions that affect exercise performance (e.g., prior exercise, muscle fatigue, and hyperthermia), and it has a rate of increase during exercise that is negatively associated with exercise tolerance [1,2,3,4,5]. The *f_R_* response during exercise differs substantially from that of tidal volume (V_T_) because the two components of minute ventilation are largely modulated by non-metabolic and metabolic inputs, respectively. As such, *f_R_* is defined as the behavioral component of minute ventilation, while V_T_ is considered the metabolic component [1,3]. The differential control of *f_R_* and V_T_ supports researchers’ and companies’ efforts to measure *f_R_* in applied exercise contexts [1].

The abundance of contact-based sensors measuring *f_R_* offers different solutions for developing wearable devices that can be used for meeting the various and challenging requirements of exercise monitoring in different applied scenarios [6,7,8]. A widely exploited method is recording respiratory-induced chest wall movements with sensors (e.g., strain sensors) that can be embedded into straps [9,10,11] or garments [12,13,14]. However, with these devices, motion artifacts can compromise the quality of the signal and with it the correct estimation of the respiratory parameters. A similar problem is faced by another method that is attracting interest in the field of sport and exercise, i.e., the extraction of *f_R_* from cardiac signals recorded from devices commonly used by athletes and exercise practitioners [15,16]. Other emerging trends in respiratory monitoring include the development of facemasks embedding sensors able to collect the respiratory waveform, and different methods can be exploited for this purpose. For instance, we have recently developed a smart facemask embedding a temperature signal and measuring *f_R_* with good precision and accuracy during cycling exercise [17]. However, this solution might be affected by external environmental conditions in some circumstances (e.g., air temperature very close to the temperature of exhaled air). A potentially interesting method that has received limited attention so far is the estimation of *f_R_* by recording respiratory sounds [18]. Anecdotally, endurance athletes monitor their opponents’ breathing sounds to gauge their physical effort during competitions [2], and microphones could be used to capture this valuable information. Microphones can easily be integrated into facemasks or other devices, and the sound signal can be processed for extracting *f_R_* [18,19,20,21]. There are different types of microphones that employ a variety of methods to convert the air pressure variations of a sound wave to an electrical signal. The most common microphones available in the market are the dynamic microphone (which uses a coil of wire suspended in a magnetic field), the condenser microphone (which uses the vibrating diaphragm as a capacitor plate), and the piezo-electric microphone (which uses a crystal of piezoelectric material) [22]. Among all of these, condenser microphones have many benefits, including high sensitivity and stable frequency response in a wide bandwidth.

The attempts made so far to extract *f_R_* from the sound signal have mostly been performed at rest and in conditions where ambient noise was experimentally minimized [23,24]. In these conditions, inhalation and exhalation show characteristic features detectable from the sound respiratory signal [25,26,27,28,29]. These characteristics in the signal are attenuated when these devices are used during exercise, where extracting *f_R_* from the breathing signal is more challenging due to breathing-unrelated sounds generated by the athlete’s movements and environmental noise, among others. As such, the microphone’s location is an important factor to consider in the exercise scenario. In the present study, we embedded a microphone in a facemask to increase the signal-to-noise ratio of the respiratory signal. This position is advantageous for two main reasons: (i) the microphone placed inside the facemask is less prone to ambient noise; (ii) if the exhaled air hits the microphone, the sensor output is enhanced. The microphone’s performance in detecting *f_R_* was evaluated at rest and during walking and running by comparing it with the *f_R_* extracted from a reference airflow signal. Given the paucity of studies performed during exercise, this study is expected to provide preliminary results on the suitability of estimating *f_R_* from a respiratory sound signal collected during exercise. 

## 2. Background and Working Principle

The microphone is a transducer that converts acoustic signal into electrical signal. It has many different applications, including voice communication, hearing aid, noise cancellation, and direction detection. In general, microphones can be classified into three major types, which are piezoelectric, capacitive, and electromagnetic-induced microphones, with different physical mechanisms to transduce sound vibrations into electrical signals. Piezoelectric microphones produce electrical signals by applying mechanical stress on piezoelectric materials. However, besides encountering the issues of high output impedance and high self-noise level, piezoelectric microphones need an external preamplifier, which does require a power source, to reduce the equivalent noise level [30,31]. In addition, a disadvantage is their low sensitivity and high noise level. The forementioned drawbacks make piezoelectric microphones less competitive than other well-developed ones [32]. A condenser microphone uses a variable parallel-plate capacitor to sense the acoustic vibration changing the distance between two plates and causing output voltage variation. With high sensitivity and good frequency response, condenser microphones have become one of the mainstream choices. Hence, we decided to use condenser microphones because of their many benefits, such as high sensitivity and stable frequency response in a wide bandwidth. In addition, these microphones are more compact and are more suitable for use with other devices (e.g., smartphones). This makes them ideal for integration into wearable devices, unlike other microphones.

In this work, two condenser microphones were used in a wearable fashion to monitor respiratory rate during exercise. One of the two microphones was embedded in a wearable facemask, while the second one was clipped on a headband. The microphone’s working principle is based on the transduction of the acoustic pressure changes into an electrical signal. The primary transduction methods in the measurement chain can be electrets, moving coils, piezoelectric elements, optical fibers, and capacitors. The last was chosen in our work due to its excellent sensitivity and accuracy, good step response time, and wide frequency bandwidth [33]. 

**Figure 1:**
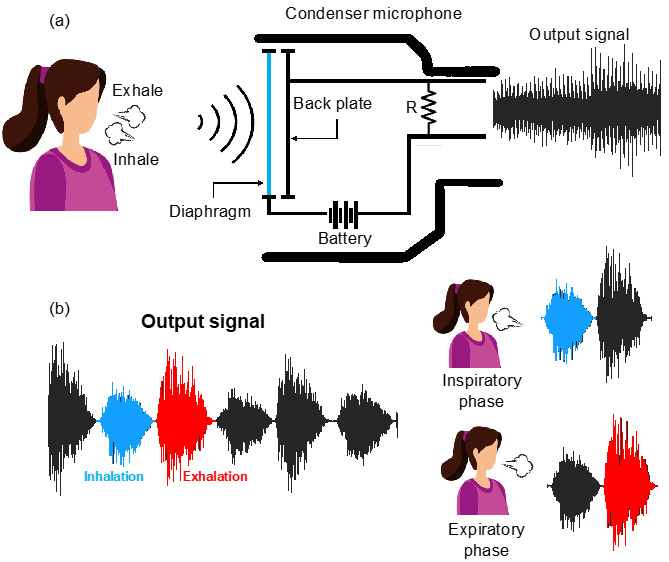
(**a**) overview of the working principle of a condenser microphone during breathing and (**b**) example of an audio signal acquired during breathing with its inhalation (in blue) and exhalation (in red) phases.

Condenser microphones have the shape of hollow metal cylinders. Inside, there is a metal foil, a metal diaphragm, and a perforated ferrule (from bottom to top). The latter does not perform any electrical function but performs the critical action of protecting the membrane from mechanical stress. The metal foil is the capacitor’s fixed plate (or back plate), and the diaphragm is the mobile plate (see Figure 1a). The diaphragm vibrates very close to the acoustic wave by which it is struck [34]. This vibrating movement causes a change in the distance between the two plates and thus a sudden change in the capacity value of the capacitor, according to Equation (1).

(1) $$C=S\cdot \frac{\varepsilon }{d}$$

where *C* is the capacitance, *S* is the effective area of the plates, ε is the dielectric constant, and *d* is the distance between the two plates. Hence, with the movement of the membrane, the variable component will be *d*, and, as a result, *C* will also be variable. The condenser microphones require the application of a voltage to polarize and distance the two condenser elements. The power supply for this type of microphone is called phantom power, a 48 V DC voltage. The change in *C* implies a difference in the output voltage *V_C_* at the ends of the capacitor and thus a change in the voltage across the electrical contacts in the microphone, as in Equation (2).

(2) $$Q=C\cdot V_{C}$$

where *Q* is the quantity of the electrical charge installed on the plates (assumed constant as the capacitor is always charged), and *V_C_* is the voltage at the end of the capacitance. Therefore, using Equation (1) in Equation (2), the output voltage and its variations contain information about changes in *d* and thus in the acoustic pressure on the diaphragm. Additional circuitry may be used to reduce electrical noise and to achieve wide dynamic ranges [35].

With regard to the application of interest, when a condenser microphone is placed near the mouth or nose, its diaphragm is struck by acoustic pressure waves generated by inhalation and exhalation [29]. Moreover, the proposed system considers a second contribution that occurs mainly during the exhalation phase: the flow of exhaled air hitting the microphone diaphragm. Although different, both phenomena (i.e., acoustic pressure waves and the airflow hitting the diaphragm) produce a change in the distance between the condenser plates and, therefore, a change in capacitance. The combination of these two contributions results in the amplification of the sensor output as the initial step of the airflow directly impacts the microphone diaphragm. The pressure waves produce vibrations in the diaphragm at characteristic frequencies ranging from 500 Hz to 5000 Hz [36]. That results in a typical output voltage signal, as shown in Figure 1b.

## 3. Experimental Tests during Walking and Running

In this section, we present the design and architecture of the wearable system for monitoring respiratory activity, the experimental protocol carried out during walking and running, and the analysis on the obtained data.

### 3.1. Experimental Setup

The experimental setup consists of a treadmill (RHC500 Treadmill, Air Machine S.r.l., Cesena, Italy) on which running tests were performed at different walking/running speeds, the proposed system for collecting audio signals, and a reference flowmeter for recording reference signals against which the proposed system has been compared.

**Figure 2:**
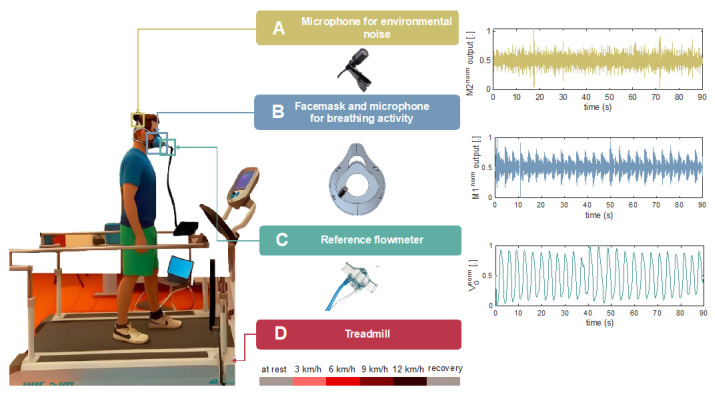
Schematic view of the experimental setup consisting of: A. a microphone (M2) used for the evaluation of the environmental noise; B. a 3D-printed facemask embedding one microphone (M1) for the monitoring of breathing activity; C. a flowmeter for collecting reference breathing signals; and D. a treadmill used to carry out the experimental tests at different walking/running speeds. The right panels provide sample output signals from each sensor used during the tests.

*(1) Proposed system:* The proposed system comprises a 3D-printed thermoplastic polyurethane wearable facemask with an embedded condenser microphone (SYNCO Lav-S6R, Guangzhou Zhiying Technology Co., Ltd, Guangzhou, China), as illustrated in Figure 2. An omnidirectional microphone (referred to as M1) was strategically positioned and embedded into the facemask to capture airflow from both the mouth and nose. The positioning was chosen to optimize the monitoring of respiratory activity and minimize the microphone’s sensitivity to ambient noise. To decrease the airflow resistance and accommodate the reference flowmeter, an anterior hole was created in the facemask. The facemask also has four housings for the headband, which the athlete can wear and adjust to ensure that the facemask fits the face correctly. A second condenser microphone (hereinafter, M2) was clipped on the headband to assess ambient noise (see Figure 2). The design was intended to meet the requirements of *f_R_* monitoring during running without restricting the athlete’s mobility or influencing the proper execution of exercise sessions.

The two microphones were powered by a 48V phantom power supply via an audio interface (AudioBox USB^®^ 96, PreSonus Audio Electronics, Los Angeles, USA), and audio signals were collected at a 44,100 Hz sampling rate. 

*(2) Reference flowmeter*: The reference respiratory pattern was recorded using a commercially available variable orifice flowmeter (SpiroQuant P from EnviteC, Honeywell, North Carolina, US) [37,38]. The flowmeter was inserted into the designated aperture of the facemask using a plastic adapter. The operating principle is based on converting the flow rate into a pressure drop between two static taps. The latter is then measured by a differential pressure sensor (163PC01D36, Honeywell, North Carolina, US) and converted into a voltage signal (V_O_ acquired at a sampling rate of 250 Hz, sensitivity 0.0069 V·min·L^−1^). A data acquisition board (DAQ NI USB-6002 from National Instrument, Texas, US) was used for both the pressure sensor power supply (+5 V supply) and the recording of the analog output V_O_. 

An example of the whole experimental setup is shown in Figure 2.

### 3.2. Experimental Protocol

Experimental tests were carried out on ten recreationally active healthy volunteers (age: 25 ± 1 year, height: 174 ± 8 cm, body mass: 69 ± 11 kg, Body Mass Index: 23 ± 2 kg/m^2^, expressed as mean ± standard deviation). The principles of the Declaration of Helsinki were followed in all steps of the study; written informed consent for study participation was obtained from all the volunteers. The study was approved by the Ethical Committee of University Campus Bio-Medico di Roma (code: 27.2(18).20 dated 15 June 2020).

Participants wore the proposed system and adjusted the headband for comfort and to ensure proper adherence to the face. The reference flowmeter was then inserted into the designated aperture of the facemask and connected to the acquisition board.

Participants were instructed to step onto the treadmill for an initial familiarization phase, followed by a 120 s warm-up period. Subsequently, they were firstly encouraged to perform a synchronization starting sequence, which involved taking three deep breaths followed by a period of apnea, and then instructed to follow the protocol, which consisted of the following phases:

- A resting phase: participants were asked to stand and breathe spontaneously for 90 s.
- A walking phase at 3 km/h followed by a 6 km/h walking phase. Each of the two stages lasted 90 s.
- A running phase at 9 km/h followed by a 12 km/h running phase. Each of the two stages lasted 90 s.
- A recovery phase in a standing position while breathing spontaneously for 90 s.

The different exercise phases were interspersed by 30-s transition periods during which participants gradually reached the speed set by the protocol.

## 4. Data Analysis

Data acquired from both the proposed system and the reference flowmeter were post-processed in a MATLAB^®^ (Mathworks, Inc.) environment. 

Prior to data analysis, all the recorded signals (i.e., M1, M2, and V_O_) were synchronized from the end of the apnea performed during the starting sequence. 

### 4.1. Flowmeter Signal Processing

**Figure 3:**
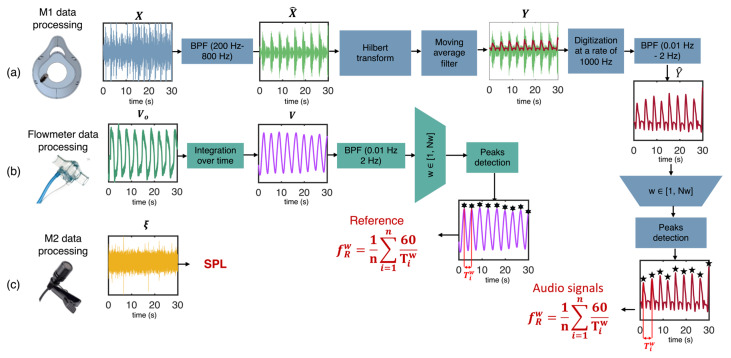
Flowchart of the data processing of audio signals for respiratory signal extraction (**a**), flowmeter output signals for reference respiratory signal extraction (**b**), and audio signals for ambient noise evaluation (**c**). BPF: third-order Butterworth band-pass filter.

The V_O_ signal of the reference flowmeter has the same behavior as a respiratory airflow. It is a pseudo-periodic signal with a negative phase corresponding to inhalation and a positive phase corresponding to exhalation (see Figure 3b). In line with previous studies, the signal was integrated over time to obtain a respiratory volume (V) signal and then normalized [37,39]. Consequently, the V signal exhibited an ascending trend during the exhalation phase and a descending trend during the inhalation phase.

The frequency content outside the breathing bandwidth was removed using a first-order Butterworth bandpass filter. Therefore, a low cut-off frequency of 0.01 Hz was used to remove slow variations in the signal unrelated to the respiratory activity. A high cut-off frequency of 2 Hz was used to remove high-frequency noise (unrelated to breathing activity), such as that produced by abrupt movements or circuit noise. 

### 4.2. Audio Signal Processing for Respiratory Rate Estimation

To emphasize these trends in the signal (X in Figure 3), a third-order Butterworth bandpass filter was applied to the audio signals collected by M1 in the 200–800 Hz range ($\hat{X}$) [40]. By using this step, we were able to eliminate high- and low-frequency noise that was not associated with breathing activity, thereby minimizing the impact of heart sounds, common 50 Hz electronic noise, and high-frequency noise.

**Figure 4:**
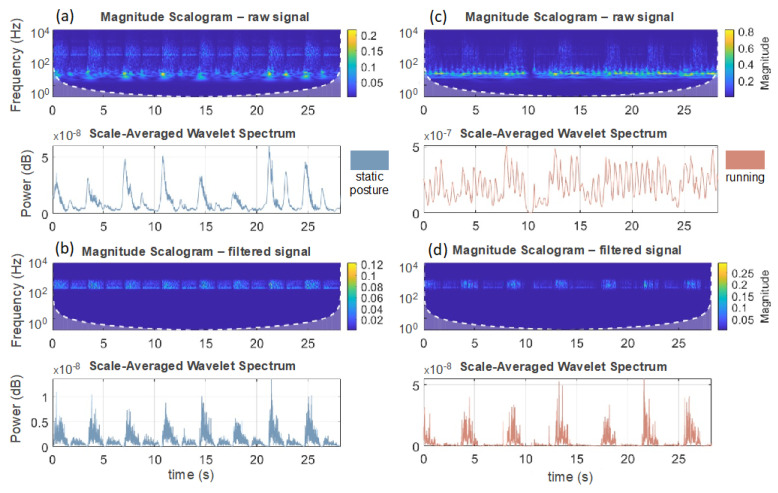
Sound scalogram of raw (**a**,**c**) and filtered (**b**,**d**) 30-s breathing signals for both static (left panels) and dynamic (right panels) tests.

Figure 4 shows the sound scalogram of raw (a, c) and filtered (b, d) 30-s breathing signals for static (a, b) and dynamic (c, d) tests. It is important to note that a higher amount of spectral power is observed within the range of 200 to 800 Hz.

The Hilbert transform was then applied to the filtered signal to obtain the audio signal’s envelope (Y) [25]. For a generic signal *x(t),* its Hilbert transform ($H\left(x\left(t\right)\right)$) is defined as in Equation (3):

(3) $$H\left(x\left(t\right)\right)=\frac{1}{\pi }\int_{-\infty }^{\infty }x\left(\tau \right)\frac{1}{t-\tau }d\tau$$

It returns the analytic signal $x=x_{r}+jx_{i}$, characterized by a real part, the original data, and an imaginary part containing the Hilbert transform. The envelope amplitude was calculated as the amplitude of the imaginary part of the Hilbert transform (see Figure 3a). The latter was finally processed with a moving average filter and digitized at 1000 Hz to reduce the overall computational burden. 

Finally, a first-order Butterworth bandpass filter with cutoff frequencies of 0.01 Hz and 2 Hz was applied to the audio signal envelope to remove frequencies outside the breathing frequency range ($\hat{Y}$) [1,41]. After this signal processing, the audio signal exhibits a distinct peak that represents the expiratory phase and a smaller peak that represents the inspiratory phase. A schematization of the whole signal processing is shown in Figure 3a.

### 4.3. Respiratory Frequency Estimation

After the signal processing, we implemented algorithms for extracting the *f_R_* from both audio and reference signals. First, we segmented both signals by using 30 s sliding windows. Then, in each window, we selected the end-expiratory peaks as breathing events (see Figure 3a,b). The breathing events (or peaks) were chosen only if they met the following requirements:

- Two consecutive peaks are selected as separate events if their distance exceeds a minimum value set at 0.7 s [42].
- Peaks are selected only if their amplitude exceeds 2% of the maximum signal amplitude.

Subsequently, the distance between consecutive breathing events ($T_{i}^{w}$) was evaluated in each window and was employed to calculate the $f_{R}$ values (i.e., $f_{R,i}^{w}$) in the w-th sliding window. Finally, the average $f_{R}^{w}$ value in each window was computed, as described in Equation (4).

(4) $$f_{R}^{w}=\frac{1}{N}\sum_{i=1}^{N}\frac{1}{T_{i}^{w}}$$

where w denotes the w-th window, and N represents the total number of breathing events selected in the w-th window. 

After the $f_{R}$ computation on both proposed and reference systems, the agreement between the $f_{R}$ values extracted from the audio signals and the reference flowmeter was evaluated in terms of mean absolute error (MAE), as in Equation (5).

(5) $$MAE=\frac{1}{N}\sum_{j=1}^{N}\left|f_{R\;device}^{j}-f_{R\;reference}^{j}\right|$$

Also, we performed the correlation plot and the Bland-Altman analysis [43] to evaluate the agreement in terms of the mean of differences (MOD) and limits of agreement (LOAs) defined as follows:

(6) $$MOD=\frac{1}{N}\sum_{j=1}^{N}f_{R\;device}^{j}-f_{R\;reference}^{j}$$

(7) $$LOA=\pm 1.96\;*\;std\left(f_{R\;device}^{j}-f_{R\;reference}^{j}\right)$$

The analysis provides insights into whether the proposed method exhibits underestimation or overestimation compared to the reference method (indicated by the MOD). Additionally, it offers an understanding of the data dispersion (represented by the LOAs) and how this dispersion varies in relation to the measured values [43]. 

### 4.4. Ambient Noise Estimation

It is known that the major artifacts in audio signals for estimating $f_{R}$ values are caused by environmental noise [25,44]. Indeed, this may cover the information on respiratory activity contained in the audio signal. To assess the influence of ambient noise on the $f_{R}$ measurement, we calculated the sound pressure level (SPL or Lp). The calculation was performed for the entire test using the microphone positioned outside the facemask (i.e., M2).

Therefore, starting from the audio signal obtained from the M2, the SPL can be determined by Equation (8):

(8) $$SPL=20\;\log_{10}\frac{p1}{p2}[dB]$$

where p1 is the sound pressure generated by the examined sound source expressed in μPa, p2 is the common sound pressure level reference point, the human hearing threshold of 20 μPa.

## 5. Results

**Figure 5:**
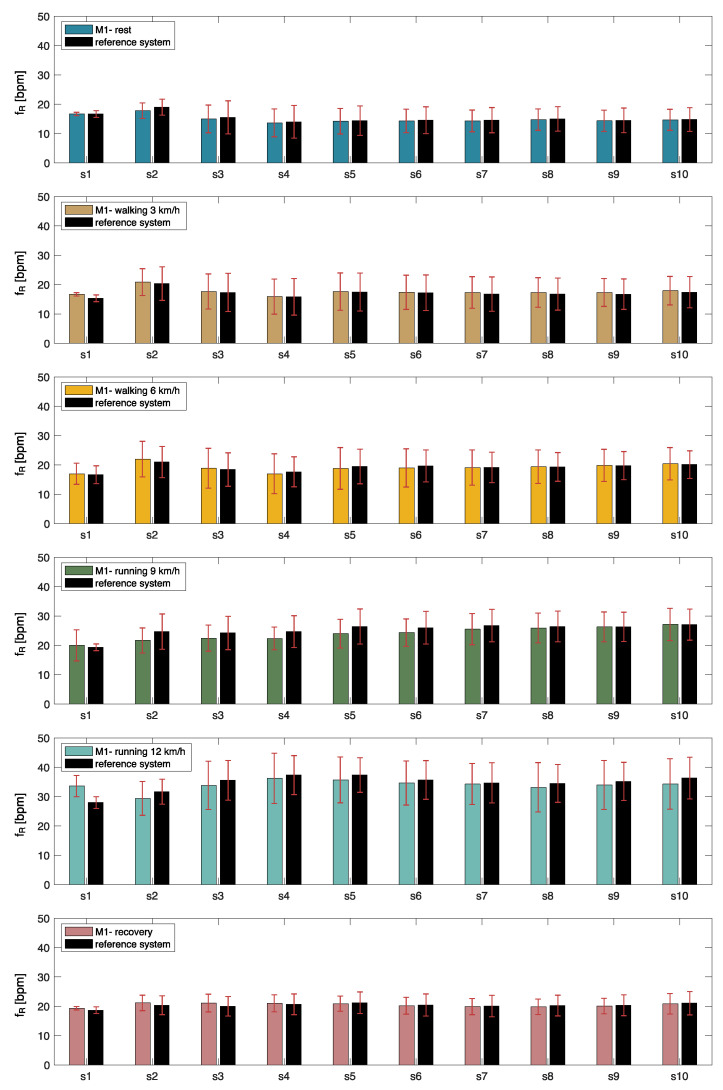
*f_R_* values calculated per participant (denoted as “s”) at the resting, walking, running, and recovery phases, expressed as mean and standard deviation.

Figure 5 shows the mean and standard deviation of the $f_{R}$ values for each physical activity level, indicating that the $f_{R}$ averaged over the volunteers ranges from ~15 bpm (i.e., breaths per minute) to ~36 bpm. Also, to compare the proposed wearable device with the reference system, we calculated the MAE values for each protocol phase (see Table 1).

Better performances were obtained in the cases of low activity levels, lower $f_{R}$ values, and during walking (max MAE value of 0.5 bpm during at-rest state at the start of the protocol and 1.5 bpm during walking). In contrast, worst performances were found when higher activity level exercises were performed by the volunteers and greater $f_{R}$ values were reached (max MAE value of 3.8 bpm during running). 

In addition, Table 1 shows the overall system performance calculated by jointly considering all tests at different activity levels and all volunteers. This condition reports an average MAE value of 1.7 bpm.

**Figure 6:**
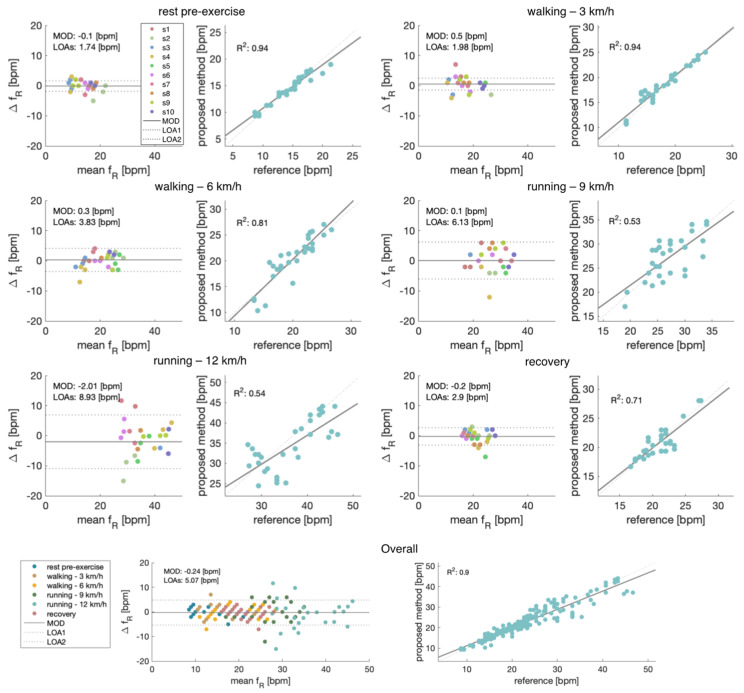
Bland-Altman plots: MOD (continuous black line) and LOAs (black dotted line) during all the protocol phases at different walking/running speeds and during rest postures. The overall configuration in which all poses were jointly considered is also shown in the bottom panels.

To deeply investigate the performance of the proposed system against a reference one in respiratory rate monitoring during running, we performed the Bland-Altman analysis and the correlation plot (see Figure 6). 

Considering the analyses carried out by looking at the different conditions separately, the *f_R_* extracted from the audio signals showed better performance when the subject was at rest in the pre-exercise condition (MOD ± LOAs: −0.1 ± 1.74 bpm) and when the participant was walking at 3 km/h (MOD ± LOAs: 0.5 ± 1.98 bpm) when compared to the performance related to the running conditions (0.1 ± 6.13 bpm for running at 9 km/h and −2.01 ± 8.93 bpm for running at 12 km/h, expressed as MOD ± LOAs). These results are supported by the correlation plot in which the R^2^ decreases with the intensity of the activity level, from 0.94 during resting at the start of the protocol up to 0.53 during running at 9 km/h. Instead, considering the system’s overall performance where all postures were jointly considered, the Bland–Altman analysis shows the MOD of −0.24 bpm and LOAs of ±5.07 bpm. The overall performance of the correlation plot shows an R^2^ value of 0.9. 

**Figure 7:**
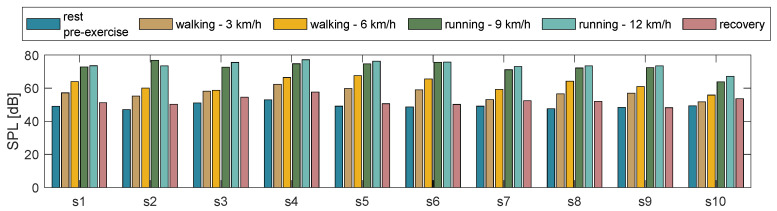
Ambient noise (SPL levels) for each participant and different conditions (resting state, walking, and running).

To assess the environmental noise during the experimental trials, we calculated the average SPL for each subject and each phase of the protocol. Figure 7 shows a bar plot where each bar represents the SPL expressed in dBs.

Figure 7 shows that for all subjects, the ambient noise increases as the intensity level of the activity increases, with SPL values ranging from 45 dB during static postures to 78 dB during running at 12 km/h. 

## 6. Discussion and Conclusions

This study evaluates the feasibility of estimating $f_{R}$ from breathing sounds during walking and running activities. While different previous studies have supported the use of microphones for respiratory monitoring by using built-in smartphones [25,45], headphones [29], or phonendoscopes [28], the use of such sensors was limited to controlled environments or stationary conditions (e.g., monitoring during sleep) [23,24,26]. It is therefore unclear whether the use of a microphone for $f_{R}$ monitoring can be extended to other everyday scenarios (such as walking or running). To address this issue, we designed a measurement system aimed at enhancing the signal-to-noise ratio of the respiratory sound signal, so that each respiratory act could be distinguished even in the presence of ambient noise (caused, for example, by running exercise, as shown in Figure 7). Hence, a condenser microphone was embedded in a facemask, which provides partial protection from external environmental noise and allows the sensor to be hit by exhaled air, thus enhancing the respiratory sound wave. The performance of our system in estimating $f_{R}$ during exercise was relatively good, and the results obtained have the potential to inform future attempts to monitor breathing with microphone sensors in the context of sports and exercise. 

Different techniques have been used in the literature to extract breathing information from audio signals [46], but only a few attempts have been made to develop portable and unobtrusive devices measuring $f_{R}$. For example, [44] proposed the estimation of $f_{R}$ in various environments using microphones embedded in facemasks. They extracted the $f_{R}$ in both indoor (office) and outdoor (public street, public bus, and subway) settings. However, they used a frequency domain approach for extracting $f_{R}$, and the low resolution of the power spectral density leads to a 0% accuracy error in most cases, making the results difficult to compare to other studies. Furthermore, many studies used built-in microphones in smartphones; [45] propose a method for real-time detection of breathing phases with a smartphone microphone placed near the mouth and the nose. When compared to a reference system, a MAE of 4 bpm in $f_{R}$ detection was found in that study. In addition, [26] used a developed acoustic sensor placed near the mouth for respiratory monitoring during sleeping. The sensor was compared with a reference system obtaining MOD ± LOAs of 0.1 ± 1.23 bpm from the Bland-Altman analysis. Nevertheless, a maximum respiratory rate of 20 bpm was achieved during the experimental protocol, and the system’s performance was not evaluated in the presence of motion or ambient noise, which is typical of an unstructured environment. Hence, the studies conducted so far differed substantially from our attempt to investigate the suitability of extracting $f_{R}$ from breathing sounds registered during exercise, as reported in Table 2.

Given anecdotal reports that the breathing sound of exercising athletes can easily be detected by teammates and opponents [2], the use of microphones for recording the breathing sound during exercise is potentially attractive, as the sensor could, in principle, be located relatively far from the mouth and nose of the athlete. However, the paucity of studies attempting to estimate $f_{R}$ using microphones during exercise required us to test a solution designed for improving the signal-to-noise ratio of the respiratory sound signal before evaluating more challenging and unobtrusive solutions. Hence, we embedded a microphone into a 3D-printed facemask for two main reasons. First, if the microphone is hit by exhaled air, the sensor output is enhanced. This is evident from the signal reported in Figure 4 that shows how the amplitude of the respiratory sound signal is much bigger during the expiratory phases than during the inspiratory phases, especially during running, which is characterized by a higher expiratory flow than during the resting condition. Hence, the flow of exhaled air hitting the microphone enhances the sensor output, which is solely dictated by the acoustic wave generated by breathing when the microphone is not hit by exhaled flow. Second, within a facemask, the microphone is expected to be partially protected from ambient noise. The comparison between the output of the two microphones used in this study supports this premise. Indeed, M1 (located in the facemask) managed to effectively record respiratory sound signals and was only partially affected by ambient noise, while M2 (clipped on the headband) essentially recorded ambient noise rather than respiratory sounds. However, we observed an increase in MAE with the increase in the sound pressure level recorded by M2 when switching from walking to running, despite the latter condition showing a greater amplitude of the respiratory sound signal. This suggests that ambient noise remains one of the primary sources of error when attempting to estimate $f_{R}$ with microphone sensors during exercise and that sensor placement is critical to obtain accurate and reliable measures [47]. Nevertheless, our results show good agreement in terms of MAE when compared to the reference system. The lowest MAE values were found at rest (0.5 bpm on average), while MAE increased during walking (0.8 bpm at 3 km/h and 1.5 bpm at 6 km/h on average) and even more during running (2.5 bpm at 9 km/h and 3.8 bpm at 12 km/h on average). The results obtained at rest and during walking are similar to those reported in previous studies using a microphone to monitor $f_{R}$ in supine position [23,48] or during sitting or standing postures [49] and even lower than those reported in a study monitoring $f_{R}$ from nasal sounds [25]. However, the error found during running, but not that found during walking, is generally larger than that observed by previous studies testing devices embedding strain sensors recording respiratory-induced chest wall movements [7,8,9,11,12]. 

Some limitations of our work should be acknowledged and addressed in future studies. The relatively small sample size tested does not allow us to generalize our findings to populations and conditions different from those tested herein. This is particularly relevant when assessing the performance of the proposed system in other environmental conditions (e.g., outdoors), given the susceptibility of the respiratory sound signal to ambient noise. Further studies should also assess the performance of the system in athletes exercising at higher exercise intensities and expiratory flow levels than the healthy individuals tested in this investigation. It would also be relevant to enlarge the testing time for assessing the feasibility of using the proposed system from a computational perspective. Indeed, we collected raw data that may increase the computational burden and noise when aiming for long-term monitoring of athletes. Hence, it would be interesting to evaluate the efficacy of audio and noise compression techniques using, for example, neural networks [50] or the methods reported in [51,52]. Microphone sensors are also suitable for addressing an important but overlooked problem when monitoring breathing in the context of exercise. It is not unusual to observe athletes or exercise practitioners talking while exercising (e.g., with a teammate). However, the act of speaking poses different challenges for monitoring breathing. From a physiological perspective, the respiratory duty cycle changes extensively (inspiratory time is considerably shorter than expiratory time), and *f*_R_ lowers [53,54,55]. Hence, it is important to identify the passages where the user talks because they contain different physiological information compared to those where he/she exercises without talking. From a measurement perspective, the identification of those passages may be more or less challenging based on how speech influences the respiratory signal of interest. Interestingly, the respiratory sound signal is highly sensitive to speech, unlike the signal recorded with other sensors (e.g., strain sensors or inertial sensors) [56]. This feature could be exploited for developing automated methods to identify and exclude speech passages with dedicated algorithms when analyzing the respiratory sound signal. Likewise, the concomitant measurement of breathing from microphones and strain sensors could be useful for testing the efficacy of algorithms identifying speech passages from signals recorded from the latter sensors. 

In conclusion, our study evaluated the feasibility of estimating $f_{R}$ from signals collected with a microphone placed at mouth and nose levels in a facemask during walking and running. Given the paucity of previous attempts to monitor breathing with microphones in the sport context, this solution was designed to increase the signal-to-noise ratio even in the presence of ambient noise. The location of the sensor allowed exhaled air to hit the microphone and enhance the sensor output, especially at higher exercise intensities, where a higher expiratory flow is expected. On the other hand, environmental noise increased when switching from walking to running and when increasing treadmill speed. As a result, the proposed system performed better during walking than running. While the accuracy and precision of the system were relatively good when evaluating all the conditions together (MOD ± LOAs of −0.24 ± 5.07 bpm), the performance observed by our system during running was generally lower than that of chest straps or smart shirts integrating strain sensors tested in previous studies [7,8,9,11,12]. Hence, the effective use of microphone sensors for estimating $f_{R}$ during exercise is critically dependent on the influence of ambient noise even when the sensor is embedded within a facemask. Future studies should identify strategies to improve the performance of microphone sensors in the presence of ambient noise or exercise modalities (e.g., race walking) and sporting environments where this issue is mitigated. Nevertheless, our findings suggest that microphone sensors can be considered among the suitable options for estimating *f_R_* during exercise.

## Figures and Tables

**Table 1:** Average (avg) $f_{R}$ values and MAE values expressed as mean ± standard deviation and calculated considering the protocol stages both separately and together (Overall).

Activity	$M1\;\mathrm{Avg}\;f_{R}$ [bpm]	$\mathrm{Reference}\;\mathrm{Avg}\;f_{R}$ [bpm]	MAE [bpm]
Rest pre-exercise	14.7 ± 3.6	14.8 ± 4.0	0.5 ± 0.4
Walking at 3 km/h	17.9 ± 4.9	17.4 ± 5.3	0.8 ± 0.6
Walking at 6 km/h	20.4 ± 5.5	20.1 ± 4.7	1.5 ± 1.2
Running at 9 km/h	27.1 ± 5.5	27.0 ± 5.3	2.5 ± 1.3
Running at 12 km/h	34.3 ± 8.6	36.3 ± 7.1	3.8 ± 2.5
Recovery	20.8 ± 3.5	21.0 ± 4.0	1.1 ± 0.7
**Overall**	**22.6 ± 8.4**	**22.8 ± 8.8**	**1.7 ± 1.2**

**Table 2:** Overview of some existing works using commercial microphones to estimate *f_R_*. MAE: mean absolute error; RMSE: root-mean-square error; DA: detection accuracy of breathing; ME: median error; SR: successful rate of the sleep RR detection; Acc: accuracy of detection; N.D.: not declared.

Work	Device (Type)	Algorithm	Study Description	Main Results
Nam et al. 2015 [25]	Smartphone microphone (MEMS—Micro-Electrical-Mechanical System—microphone)	Autoregression	Tracheal and nasal breathing in an office	ME: 1%
Kumar et al. 2021 [29]	Headphones microphone (MEMS microphone)	LSTM	Workout in both indoor and outdoor environments	DA: 66%
Ahmed et al. 2023 [24]	Earbuds microphone (MEMS microphone)	random forest, MLP	Sitting, standing, and lying in both lab and at home tests	MAE: 1.36 bpm
Abbasi et al. 2018 [26]	Dedicated body-mounted microphone (Capacitor microphone)	N.D.	Mouth and nasal sounds when lying down	RMSE: 1.26 bpm
Fang et al. 2018 [23]	Wireless headset microphone (N.D.)	Peak detection	Mouth and nasal sounds during sleep	SR: 98.4%
Skalicky et al. 2021 [28]	Phonendoscope Littmann 3200 (N.D.)	Transition between inspiratory and expiratory phases detection	Lung sounds while standing	Acc: 0.2 s
Shih et al. 2019 [45]	Smartphone microphone (MEMS—MicroElectrical-Mechanical System—microphone)	LSTM, CNN	detection of breathing phases during normal chest breathing and deep abdominal breathing	MAE: 4 bpm
**Our study**	**Facemask-mounted** **microphone** **(Capacitor microphone)**	**Peak detection in the time domain**	**Mouth and nasal sound during walking and running**	**MAE: 1.7 bpm**

## Data Availability

The data presented in this study are available on request from the corresponding author. The data are not publicly available due to privacy reasons.
